# Land Tenure, Ownership and Use as Barriers to Coastal Wetland Restoration Projects in Australia: Recommendations and Solutions

**DOI:** 10.1007/s00267-023-01817-w

**Published:** 2023-04-03

**Authors:** Justine Bell-James, James A. Fitzsimons, Catherine E. Lovelock

**Affiliations:** 1 TC Beirne School of Law, University of Queensland, Brisbane, QLD Australia; 2 The Nature Conservancy, Carlton, VIC Australia; 3 School of Life and Environmental Sciences, Deakin University, Burwood, VIC Australia; 4 School of Biological Sciences, University of Queensland, Brisbane, QLD Australia

## Abstract

Globally, there is an urgent need for widespread restoration of coastal wetlands like mangroves and saltmarsh. This restoration has been slow to progress in Australia for a number of reasons, including legal issues surrounding land tenure, ownership and use. This paper uses the responses to a survey of coastal zone experts to identify and articulate these legal issues, before considering and analysing in-depth recommendations, solutions and levers to facilitate restoration, and areas where further research or possible policy and/or law reform is needed. It calls for legislative reform to clarify tidal boundaries generally and under sea-level rise, greater use of incentive schemes to encourage the uptake of restoration projects, and utilisation of contracts and land-based covenants to secure projects and carbon flows.

## Introduction

It is now well known that coastal wetlands (including saltmarsh and mangroves) deliver crucial ecosystem services like water filtration, carbon sequestration, fisheries habitat and shoreline protection (see e.g. Barbier et al. 2011; Costanza et al. 2014). However, these marine and estuarine ecosystems have been historically undervalued, with between 30-50% of global coastal wetland extent lost during the 20^th^ century (see e.g. Friess et al. 2019). This loss has occurred at higher rates than terrestrial forests (Ramsar Convention on Wetlands, 2018), and mostly before the true value of these coastal ecosystems were realised (see e.g. Li et al. 2018). As the importance of these ecosystems has become better appreciated, the rate of mangrove loss in particular has declined due to improved conservation efforts (Friess et al. 2020). However coastal wetlands continue to be threatened by activities and phenomena including clearing and development, aquaculture, fishing, pollution and climate change (Friess et al. 2020; Gedan et al. 2009; Halpern et al. 2008; Unsworth et al. 2019). While action to mitigate these future threatening processes must be paramount in government policy approaches, there is also an urgent need for widespread restoration of past harm in the coastal zone (Bayraktarov et al. 2016; Bell-James et al. 2022; Friess et al. 2020; Murray et al. 2022; Saunders et al. 2020) and planning to allow for the inland migration of coastal wetland ecosystems with sea level rise (Leo et al. 2019; Mills et al. 2016).

Global momentum towards widespread ecosystem restoration is increasing (Armitage et al. 2021). The United Nations has declared the years 2021–2030 as the ‘Decade on Ecosystem Restoration’, with the aim of ‘supporting and scaling up efforts to prevent, halt and reverse the degradation of ecosystems worldwide’ (United Nations 2019, art 1). The recent Convention on Biological Diversity’s Kunming-Montreal Global Biodiversity Framework has set a target of ‘ensur[ing] that by 2030 at least 30 per cent of areas of degraded terrestrial, inland water, and coastal and marine ecosystems are under effective restoration, in order to enhance biodiversity and ecosystem functions and services, ecological integrity and connectivity’ (Convention on Biological Diversity 2022).

Despite this global momentum and increasing recognition of the importance of restoration as a crucial management tool for marine ecosystems (Saunders et al. 2020), marine restoration projects are yet to be undertaken at a large scale in Australia (Gillies et al. 2015; Saunders et al. 2022; Waltham et al. 2020), although there are some promising examples of major restoration projects that have been implemented (see e.g. Glamore et al. 2021). The reasons for this are multifactorial, including the cost of implementing projects (Bayraktarov et al. 2016), and the lack of dedicated and fit-for-purpose restoration policy, requiring proponents to engage with time-consuming and expensive processes designed for permitting development and other harmful activities (Shumway et al. 2021). Legal and policy constraints regarding land tenure, ownership and land use have also been identified as a barrier (see e.g. Bell-James and Lovelock 2019a, 2019b; Evans 2018; Saunders et al. 2022), with particular difficulties arising in the intertidal zone, as it is a legally contested space due to the intersection of multiple tenures (Friess et al. 2016; Rog and Cook 2017). This is problematic as security of tenure has been identified internationally as an important pre-cursor to restoration (see e.g. Chazdon et al. 2017), and the Society for Ecological Restoration’s Standards of Practice specifically recognise that restoration plans should ‘identify site-tenure security to enable long-term restoration and allow appropriate ongoing access for monitoring and management’ (Gann et al. 2019, p. S23). These issues combined can affect restoration projects in terms of both initial uptake and ultimate success (Evans 2018; Saunders et al. 2022).

In 2020, we conducted a broad survey of a small group of coastal practitioners, scientists and policy makers in Australia to obtain their views on the barriers to protection, management and restoration of coastal wetlands and the ecosystem services they provide, specifically from a legal perspective. These experts identified a number of key barriers spanning across several themes, and, consistent with the literature, issues associated with land tenure, ownership and use emerged as one of the major challenges. In order to drive future research, we sought to understand respondent’s views as to why these challenges exist and we found that concerns fell broadly into three categories: (a) ambiguity in determining where the boundary lies between seaward and landward areas, with restoration becoming even more complex when the wetland/s being rehabilitated spans multiple tenure types (e.g. public and private), (b) legal and policy constraints regarding land tenure, ownership and use impeding the landward migration of wetlands, and (c) the ease of restoration on public vs private land tenures. Overall, the interview responses highlighted a need for further work to examine how to navigate land tenure, ownership and use aspects of restoration projects in the intertidal zone.

In this paper we aim to consider and analyse these issues associated with land tenure, ownership and use in coastal restoration. We do not purport to present a detailed quantitative or qualitative analysis of the interview results, but rather use the themes distilled from the interviews to compile a list of issues, according to which we structure our analysis and discussion. We also seek to highlight potential recommendations, solutions and levers to facilitate restoration where they are presently available, and also identify and clarify a future agenda for further research or possible policy and/or law reform.

## Why is Land Tenure, Ownership and Use a Barrier to Coastal Restoration?

In 2020, we conducted interviews with a small group of coastal zone experts in Australia (pursuant to an ethics approval issued by the University of Queensland Business, Economics and Law Low and Negligible Risk Ethics Sub-Committee, Approval number 2019002449), including representation from government departments, non-government organisations (NGOs) and academia/research organisations. We used personal networks and government department enquiry forms to recruit 16 experts from across all Australian jurisdictions (with the exception of the Australian Capital Territory, as it has no coastal wetlands, and Western Australia, from which we were unable to recruit anyone). We sought to recruit experts who are presently employed in a role where they are directly involved with the protection, management and/or restoration of coastal wetlands. The full results of these interviews are published in Bell-James et al. (2023).

We conducted a first round of interviews with a single open-ended question:

In your opinion, what (if any) are the current legal barriers to effective management, protection and restoration of mangroves and other [coastal] wetlands?

We conducted these interviews in person or by phone in February 2020, and they were recorded, transcribed and anonymised. The transcripts were then thematically analysed (see e.g. Braun and Clarke 2006) using manual techniques. Land tenure, ownership and use issues were raised by 11 out of 16 experts in our first round of interviews and was therefore identified as one of the key themes.

To interrogate key themes in more detail we designed a more targeted survey consisting of 12 questions to be put to all informants from the first round. These questions were a mix of ‘yes/no’ and open-ended response questions, and informants were invited to write out their responses or provide them over the phone to be transcribed. Two of these questions concerned land tenure:

Q6: Is wetland protection/management/restoration easier on public or private land?

Q7: Do you have any additional thoughts on changes needed to land tenure arrangements to facilitate [coastal] wetland protection/management/restoration?

Again, survey responses were manually thematically analysed. Our manual thematic analysis of both rounds of interviews revealed that land tenure, ownership and use concerns could be grouped into three sub-themes.

First, boundary ambiguity was identified by some respondents as a problem, because it is not always easy to discern where the line between tenures (e.g. privately owned land and public land) exists (R9, R11). The second related sub-theme was that this ambiguity is particularly acute when complicated by future climate change and sea level rise and likely inland migration of coastal wetlands (R9, R11), as well as the more short-term problem of eroding banks changing legal boundaries by virtue of the operation of the doctrine of erosion (R13) (that is, the proposition that the legal boundary shifts landward or seaward with erosion or accretion). Some informants also emphasised that coastal wetlands are not static (R2) and it is important that decision-making acknowledges this (R14). In turn, this would require planning to enable the natural landward migration of ecosystems (R14, R15), although it might be complicated to change land ownership arrangements and boundary rules due to entrenched thinking about private property (R11).

The third sub-theme was that different tenure types – in particular, public versus private land – may involve particular complexities. Several respondents also stressed the importance of involving Indigenous communities in decision-making processes (e.g. R2, R3, R5). There was a general perception that protection or restoration activities can be more straightforward on public land as these activities are more likely to align with public land management goals (R7, R13, R16), and indeed in response to a targeted question in the second round of interviews most respondents stated that restoration is easier on public land (75%). Some respondents elaborated on their answer and emphasised that, on public land, the goals of the proponent and the regulator were one and the same, thus streamlining the process (R4, R14, R16). Private land projects can be impacted by changed circumstances or priorities of the landholder (e.g. a transfer of ownership, changing land management practices, financial pressures) (R6). Government ownership of land was also seen to remove the need for landholder consent for projects (R11), and purchasing private land and transferring it into public ownership could therefore negate the need for consents to be obtained (R16). However, difficulties may still arise where an activity is proposed on public land that abuts private land, as consent must be obtained from adjoining landholders because some restoration activities may have impacts on their properties (R7, R13, R16).

However, some respondents suggested that restoration projects on private land can be effective and produce good outcomes, provided that the landholder has personal values which are aligned with conservation and adequate financial resources at their disposal (R4, R12, R16). These projects could also deliver effective outcomes if incentives are provided to require landholders to protect or manage coastal wetlands, often paired with an agreement (e.g. conservation covenant) registered on land title to compel landholders to protect or manage them (R5), or supplemented by government investment in improved financial mechanisms and incentives to encourage management and restoration activities (R9).

Some respondents expressed a view that wetland protection, management and restoration projects are easier on private land. One respondent suggested that projects on public land can be harder as government agencies are possibly more risk averse than individuals, and doing nothing is often viewed as a less risky management strategy (R8). The need for consultation with community for public projects could also complicate the delivery of projects (R11). Provided that a private landholder has necessary expertise, knowledge and ability to carry out a restoration project – for example, where land is purchased by a private environmental NGO – then it was suggested that restoration can be more easily streamlined without the need to coordinate government agencies (R12). It was also noted by R11 that the most difficult situations arise when a wetland being rehabilitated spans both public and private lands. R14 also acknowledged the difficulties that may arise from the dynamic nature of coastal wetlands especially under sea-level rise, as legislation (and policy) is designed to protect wetlands in their current location.

The responses to these interviews are consistent with the broader literature that land tenure, ownership and use is a complicating factor in coastal restoration, and offer some additional insights as to what the specific complexities are. In the following sections we seek to analyse and discuss these complexities, with particular attention to any possible solutions, enablers, or areas where law and policy reform are needed.

## The Boundary between Public and Private Land can be Ambiguous and may be Impacted by Erosion and Sea-level Rise

Restoration of coastal wetlands is complicated as it is not always clear whether they are located on public land, private land, or span across both, due to boundary ambiguity (Rog and Cook 2017; Victorian Environmental Assessment Council 2020). This was a point raised in some reponses to our interviews. Coastal wetlands are located in the intertidal zone with mangroves located at approximately mean sea level to the level of the highest astronomical tide (HAT) (although they can occur higher and lower than these tidal planes in some settings), and saltmarshes in the upper intertidal zone (Rogers et al. 2016). The intertidal zone is the interface between land and sea, and traditionally in Australia the legal boundary between land and tidal water has been deliniated by a tidal reference – generally the mean high-water mark (which is below HAT), or sometimes the low-water mark. For this reason, coastal wetlands often straddle the public/private land ownership divide. Additionally, in some places the entire intertidal zone has been granted to traditional owners (e.g. in the Northern Territory by virtue of *Northern Territory v Arnhem Land Aboriginal Land Trust* (2008) 236 Commonwealth Law Reports 24).

**Fig. 1:**
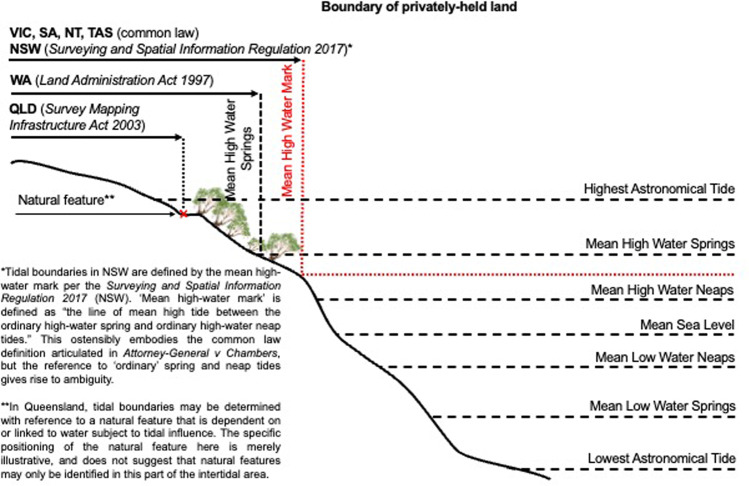
Tidal boundaries in Australia. VIC Victoria, SA South Australia, NT Northern Territory, TAS Tasmania, NSW New South Wales, WA Western Australia, QLD Queensland

With boundary ambiguity identified as a barrier to coastal wetland retoration in the literature, we sought to analyse the precise causes of this identified tidal boundary ambiguity. We found that this lack of clarity stems from several factors. First, there are jurisdictional variances in how the boundary between land and water is ascertained. Most Australian state jurisdictions continue to use the mean high-water mark as the legal boundary, although Western Australia uses mean high water springs, and Queensland uses an identified natural feature (Fig. 1; also see Bell-James and Lovelock 2019b for a detailed explanation of the Queensland approach). In some states the boundary reference may differ from parcel to parcel – for example in Victoria some ambulatory boundaries are defined by reference to the high-water mark, some the low-water mark, and some by descriptors e.g. ‘Shore of Port Phillip Bay’ or ‘on the shore of Bass Strait and the Southern Ocean’ (Victorian Environmental Assessment Council 2020, p. 39). Furthermore, there are some variances in state legislation as to how the mean high-water mark is measured and determined (Rog and Cook 2017) so even those States that use mean high-water mark are not necessarily consistent in their approach. This may create uncertainty for proponents working across multiple jurisdictions, and raises additional complexities if an ecosystem spans across jurisdictional boundaries. It could arguably also sway investment decisions to be made in jurisdictions where boundary rules provide greater certainty regarding entitlements, ownership, and potential changes thereto, rather than decisions being made based on the biophysical suitability of restoration project sites.

Second, determining the mean high- or low-water mark, springs, or natural feature (ambulatory boundary) pursuant to the rules in the relevant jurisdiction will likely require expert advice from a surveyor or geomorphologist. This is because the line may not be easily discerned by simply consulting a map, or may have changed through coastal fluctuations since any previous determination occurred (see e.g. Victorian Environmental Assessment Council 2020). This need for expert advice is an added expense in potential marine and coastal restoration projects, thereby adding a burden onto potential investors and proponents.

Third, the physical boundary between land and tidal waterways is ‘fuzzy and dynamic’ (Friess et al. 2016) and the legal boundary is ambulatory in nature, shifting landward or seaward with erosion and accretion, respectively (see discussion in Bell-James and Lovelock 2019b). The physical location of the boundary may also shift with climate change-induced sea level rise, although the legal consequences have been the subject of some debate. Traditionally, the doctrines of erosion and accretion, which provide that the boundary of land will shift either landward or seaward with erosion or accretion, would apply provided the process of change is natural, gradual and imperceptible (*Ward v The Queen* (1980) 142 Commonwealth Law Reports 308, 337; *Hazlett v Presnell* (1982) 149 Commonwealth Law Reports 107, 117). The doctrines are underpinned by a sense of ‘give and take’ in that a landholder may lose part of their land or gain additional land depending on the whims of nature, with a High Court Justice remarking that the doctrine ‘must work both ways, if at all’ (*Williams v Booth* (1910) 10 Commonwealth Law Reports 341 at 361). They also reflect the uncertain and unpredictable nature of coastal shorelines. However, the continuing appropriateness of these doctrines under climate change has been challenged by legal scholars (Bell 2014; Byrne 2012; Flournoy 2017; Michael 2018) and coastal experts (Victorian Environmental Assessment Council 2020). There are several arguments as to why the doctrines should not apply to climate change-induced sea level rise: the impacts of climate change are human-caused rather than natural, the impacts are predictable and can be estimated within a range of certainty, and climate change will remove the ‘give and take’ element as it is almost certain to result in a loss of land for the landward owner (Bell 2014; Byrne 2012; Flournoy 2017; Michael 2018). However without legal reform or court challenge (Victorian Environmental Assessment Council 2020) to alter the status quo it seems likely that tidal boundaries will remain ambulatory in nature and therefore may be altered significantly with climate change. In some cases, depending on coastal geomorphology, this could result in an entire parcel of land being inundated. This would then raise the issue as to whether a land title (or the part of the title inundated) should consequently be cancelled, and the political – if not strictly legal – question of whether compensation should be paid by government (Bell 2014). Any impacts to freehold land are also likely to trigger legal action (Victorian Environmental Assessment Council 2020).

Fourth, as Fig. 1 demonstrates, it is not unusual for a coastal wetland to span both sides of the mean high water mark, which means that a wetland may be located partly on privately owned or managed land (e.g. freehold or leasehold) and partly within a waterway that is under public ownership and control. This would raise complications for a proposed restoration project as different agencies may have management responsibility for the landward and seaward parts of a site (Rog and Cook 2017), and different permits and approvals may also be needed for these parts of the site (i.e. for the private land component and the public land component). This could potentially also mean twice the transaction costs and inputs to satisfy requirements for permits over both areas of land/water. It would also require the owners/entities in charge of all land parcels/areas to agree to the establishment of the restoration project and have the same management objectives for the wetland. There may also be a need to enter into contractual obligations to govern the terms of the project. If a project is being carried out to obtain an incentive such as carbon credits (e.g. under Australia’s Emissions Reduction Fund), it will also be necessary to determine where ownership of carbon credits should lie, which would technically require an assessment of precisely where coastal vegetation is located vis-à-vis the boundary (see e.g. Bell-James and Lovelock 2019a). However this problem can potentially be resolved contractually by agreement between the landward owner or lessee and the state, and this is the approach that has been adopted in Australian under the new blue carbon methodology (Clean Energy Regulator 2022). Therefore, there is some guidance available for overcoming issues stemming from boundary ambiguity.

## Tenure, Ownership and Land Use may Impede Landward Migration of Coastal Wetlands

The challenge of ensuring space for landward migration of coastal wetlands under climate change was a recurrent theme in both our interview responses and the academic literature (see e.g. Mills et al. 2016; Rogers et al. 2014; Schuerch et al. 2018). Climate change is causing global mean sea levels to rise at a current rate of approximately 3 mm per year, with this rate accelerating annually (Nerem et al. 2018). The most recent IPCC Report concluded that it is virtually certain that global mean sea level will continue to rise throughout the 21^st^ century, with a likely rise of between 0.38–0.77 m by 2100 (relative to the period 1995–2014) (Masson-Delmotte et al. 2021).

Coastal wetland ecosystems like mangroves and saltmarsh thrive in the intertidal zone where they are subject to periodic inundation by the tides (Rogers 2021). As sea levels rise, areas where wetlands are currently located will become subject to higher rates of inundation, potentially rendering these areas unsuitable for continued occupation. These ecosystems may have the ability to adapt to sea level change either horizontally and/or vertically; that is, they can shift inland to a higher location within the coast, or increase sediment to accrete vertically (Krauss et al. 2014). However, this ability to adapt may be affected by several factors. First, if the pace of sea level rise is greater than the pace at which wetlands can migrate or accrete, they may become submerged over time (see e.g. Jankowski et al. 2017). Second, if physical development or hard coastal defence structures prevent the landward migration of wetlands, they may be ‘squeezed’ out of the landscape in a phenomena known as coastal squeeze (Doody 2013; Pontee 2013). Providing space for inland wetland migration therefore must be a priority for government and policy-makers (see e.g. Leo et al. 2019).

Some Australian jurisdictions have addressed the potential for inland migration of coastal wetlands within their planning law frameworks which govern future development. In Tasmania, land may be zoned as ‘future coastal refugia’, defined as ‘land where coastal processes are likely to occur naturally and can continue to occur, including the landward transgression of sand dunes, wetlands, saltmarshes, and other sensitive coastal habitats due to sea-level rise’. Once land is zoned as such, development is controlled and must allow for natural coastal processes to occur (Tasmanian Planning Scheme—State Planning Provisions (2017) C7.3-7.6). Similarly, in New South Wales land may be identified as the ‘proximity area for coastal wetlands’, and development consent must not be granted in that area unless it will not have significant impacts on those coastal wetlands (State Environmental Planning Policy (Coastal Management) 2018, c 11).

Whilst this zoning of areas for future migration is a positive phenomenon in planning law, planning laws do not operate retrospectively and an existing lawful use of land cannot be made unlawful through the passage of new planning law (see e.g. Bell 2014). This may be problematic where potential future migration areas are held as freehold (privately owned) or leasehold (leasehold meaning land owned and leased by the State to an individual, usually for multiple decades) and the current use of that land is incompatible with landward migration of wetlands; for example, there may be a fence in place to control livestock and facilitate agricultural production (see e.g. Bell-James et al. 2022). An analysis of the underlying land tenure of current and future wetland extent showed major variations across Australian jurisdictions, but in many instances the areas where wetlands will need to migrate further into to keep pace with sea-level rise are currently zoned as freehold or leasehold land (Victorian Environmental Assessment Council 2020; Whitt 2020). For example, in NSW, currently around ~30% of coastal wetlands are located on freehold land. Under a high future emissions scenario (RCP8.5 based on the 2013 IPCC scenarios: Stocker et al. 2014), by 2100 this will be closer to ~60% (Whitt 2020). In Queensland, approximately ~70% of current extent is found on freehold or leasehold land. This will increase to around ~90% by 2100 under RCP8.5 (Whitt 2020). However, these figures are contingent on there being no impediment to wetlands migrating accordingly, which is not the case in a number of areas. If there are no physical impediments to inland migration, the boundary may shift landward pursuant to the doctrine of erosion, although as discussed above, the appropriateness of this doctrine under sea level rise remains questionable. Governments could also explore the potential to compulsorily acquire privately owned land that is considered essential for inland migration of coastal wetlands and remove any physical barriers thereto, although the costs may be prohibitive (see e.g. Mills et al. 2016), or implement other incentive schemes as discussed below.

## Difficulties with Restoration Projects on Public and Private Land

The responses to our interviews highlighted tenure type as an issue in coastal wetland restoration, with a perception amongst some respondents that restoration projects may be easier on public land (versus freehold tenure/private land) due to the proponent and the regulator being the same entity. This may be due to either ease of permitting, alignment of the objectives of the regulator and proponent, or a combination of both. Some respondents suggested that the potential need to incentivise or subsidise the uptake of projects on private land, as well as possible reluctance of private landholders to abandon a lawful use, made restoration on public land easier. We sought to interrogate this issue in greater detail to consider the barriers to restoration on private land vis-à-vis public land that may make restoration projects more difficult or more expensive.

### Permitting Regimes may Favour Public Land and Public Entities

We investigated whether it is easier (a) to obtain permits for work on public land as compared to private land, and (b) for government entities to obtain permits for restoration projects as compared to private entities (if indeed government entities need to at all). We undertook this investigation by considering the permitting requirements for a variety of restoration project types, with specific reference to distictions between proponent identity and land tenure type. To narrow our analysis we used the list of restoration activity types in Shumway et al. (2021) and considered permitting requirements for three types of restoration projects: (a) removal of a waterway barrier, or restoring hydrology, (b) revegetation and (c) removing vegetation (e.g. propagules or seedlings for transplant). The results of this legislative review are available as supplementary material.

Overall, we did not find a significant difference in permitting requirements for public entities and public lands in comparison to private entities and private land. Indeed, most legislation we considered specifically stated that it bound the jurisdictional government (the Crown), and therefore the same permits would need to be obtained by public entities for projects on public land. For example, South Australia’s *Native Vegetation Act 1991* states at the outset that it binds the Crown (s 5). It allows an owner of land to apply for consent to clear vegetation (s 28), with ‘owner’ defined to include fee simple landholders, Crown lessees, and in the case of any other land, the Minister responsible for the care thereof (s 3). This essentially covers the field of proponents and tenure types and ensures that a consent is required irrespective of tenure.

There are some examples in which it is easier for government entites to obtain permission for restoration projects. For example, in Queensland tidal works are defined as works in, on or above land under tidal water, and include construction and demolition of seawalls and embankments (*Coastal Protection and Management Act 1995* (Qld) Schedule). Where tidal works are undertaken by a local government, they are considered ‘accepted development’ and no permit is required. For other proponents, development approval is required (see *Planning Regulation 2017* (Qld)). In South Australia, the *Coast Protection Act 1972* (s 21A) provides that the Coast Protection Board is authorised to remove sand and other material from one part of the coast (not being private land) to another part of the coast for the purpose of protecting, restoring and developing any part of the coast. However this power is quite limited and will likely only apply to urgent stabilisation works.

Other factors notwithstanding, there does not seem to be a significant difference in permitting requirements sufficient to conclude that restoration projects are easier on public land. It is possible that the perception that restoration is easier on public land is not due to anything inherent in the permitting requirements or the underpinning legislation, but rather to the entity responsible for managing that tenure. It may be easier to convince a government entity to utilise state lands for restoration than it would be to convince a private landholder to do the same. If a restoration agenda is driven by government and land is owned and controlled by government, the interests of the regulator and regulated may be one and the same and the project may be streamlined (recognising that different Departments of a government may have conflicting policy objectives). The solution may therefore not just be in changing permitting requirements for private land (although permitting requirements for restoration generally on any tenure type are onerous and should be reformed (Saunders et al. 2022; Shumway et al. 2021), but rather in taking steps to better align the interests of both the government/proponent and the private landholder in order to undertake a project on private land.

### Projects on Private Land may be Impacted by a Private Landholder’s Changed Priorities or Circumstances

Some responses to our interviews also suggested that restoration projects on privately owned land by private entities are less secure as they can be impacted by changed circumstances or priorities (e.g. a transfer of ownership, changing land management practices, financial pressures). That is, even if a private landholder is initially willing to conduct or allow restoration activities on their land, the situation may change. There are certainly mechanisms that can be used to alleviate this issue, such as the purchase of private land and transfer to public ownership. However, the costs of doing so may be prohibitive (see e.g. Mills et al. 2016), would typically either require a landowner willing to sell, or use of a compulsory acquisition process which may be politically challenging to do (FAO Land Tenure Studies 2008). It might also prevent land from being beneficially used in the short- to medium-term (e.g for agriculture) in circumstances where it is not required for restoration activities or landward wetland migration until the long-term (see e.g. Byrne 2012).

As an alternative, there are also legal mechanisms available to meet these concerns whilst allowing property to remain in private ownership, such as securing the project through a legally binding instrument registered over land in perpetuity. If the purpose of the project is to create or enhance carbon stocks, all Australian states have enacted or amended legislation to provide for registrable or recordable carbon rights over the carbon stored in vegetation on freehold land,^1^ with these provisions already widely used for terrestrial vegetation projects. A carbon right would be an appropriate instrument to use to ensure that carbon stocks are legally protected irrespective of changes of ownership. Alternatively, in all Australian jurisdictions covenants can also be registered over land to document and enforce restrictions and obligations on land use (see Bell-James et al. 2022 for a detailed analysis). There is emerging research to suggest these covenants can be used on a ‘rolling’ basis similar to rolling easements in the United States (e.g. Titus 2011), which would ensure that land not being restored can remain in use (e.g. for agriculture and productive in the short- to medium-term (Bell-James et al. 2022). Voluntary but binding agreements that seek to achieve both carbon and biodiversity outcomes are increasingly being utilised in Australia (see e.g. DAWE 2022).

In summary, there are a range of legal instruments available to government to secure restoration projects on private land and ensure that the land remains available for restoration, despite change of ownership or circumstance of the landholder.

### Existing Uses of Land may be Incompatible with Protection, Management and Restoration of Coastal Wetlands

Whilst the above paragraph considers the situation where a landholder is initially willing to conduct restoration activities on their land and then changes their approach, there will also be circumstances where a landholder is not willing to voluntarily restore wetlands or allow for their landward migration at all. A major challenge is therefore that existing uses of land on freehold and leasehold tenures may be incompatible with the protection, management, restoration and landward migration of coastal wetlands. This applies to both active and passive activities: a landholder cannot be compelled to abandon existing land use and undertake active restoration activities. Further the existing use of the land and structures thereon may physically impede the future inland migration of wetlands.

Internationally, private property rights have been observed as an impediment to the delivery of ecosystem services unless a landholder possesses place attachment or is motivated to protect the environment (Bastian et al. 2017). Suggested solutions include incentive schemes (see e.g. Pease et al. 1997; Sorice et al. 2011; Welsh et al. 2018), more explicit recognition of ecosystem services in conservation covenanting legislation (Archibald et al. 2021), measures to increase landholder awareness of the importance of ecosystem services (Mikša et al. 2020), and increased public participation to assist with overcoming opposition and increasing uptake of land use change schemes (Rouillard et al. 2014).

The possible use of incentives to encourage restoration was raised in our interviews. Incentives have been used in numerous contexts to motivate land use changes and increase the supply of ecosystem services, including in Australia (see e.g. Bryan 2013; Fitzsimons 2015; Keenan et al. 2019; Zammit 2013). The use of ‘rolling covenants’ in Australia through a mechanism to restrict land use subject to inundation through sea level rise in the long-term whilst allowing uses such as agriculture on parts of land in the short- to medium-term was considered to have the best chance of success if associated with financial incentives (Bell-James et al. 2022). Whilst incentives will not be sufficient in every circumstance to convince a landholder to change a currently permissible use of land to enable coastal wetland restoration, they can facilitate some restoration on private land (McCristal 2015).

## Conclusion and Recommendations

Widespread restoration of coastal ecosystems will be required over the coming decades to ensure their continued provision of ecosystem services. There will also need to be sufficient land set aside to ensure space for the inland migration of coastal wetlands with sea-level rise. Our surveys with restoration experts shed light on the challenges inherent in the existing arrangements surrounding land tenure, ownership and use. We have identified and analysed these key challenges and make the following observations, recommendations, and identification of areas for future policy and law reform and research:

1. The array of different approaches to determining an ambulatory boundary in Australian jurisdictions (e.g mean high water mark vs natural feature) is confusing, and there should be further consideration as to whether harmonised legislative rules could provide clarity. This is especially important where coastal wetlands span across jurisdictional boundaries;
2. Determining the boundary between land and water is complex, and may be difficult to determine with certainty, especially taking into account tidal variations that may occur over the lifespan of a restoration project. Where possible, boundary ambiguity and legal rights (including rights of traditional owners) should be resolved through contractual arrangements prior to the commencement of a restoration project, such as is required under Australia’s blue carbon methodology (Clean Energy Regulator 2022);
3. The impact of sea-level rise on ambulatory boundaries remains uncertain. Further research is needed to determine the most equitable solution, and the uncertainty should also be resolved via legislative reform;
4. Implementing schemes to facilitate, encourage and incentivise restoration projects, changes of land use and space for inland migration of coastal wetlands on privately owned land should be a priority of governments;
5. These schemes should be supplemented by robust legal arrangements to secure land undergoing coastal wetland restoration in perpetuity, for example through a covenant registered on land title, and incentives prioritising such mechanisms. Novel mechanisms such as rolling covenants should be explored;
6. Whilst there are no significant distinctions in terms of permitting requirements for restoration projects on public land as compared to private land, both require interaction with legislative regimes that are complex, onerous and expensive. Reform of legislative requirements for restoration projects should also be a priority for governments (see e.g. Saunders et al. 2022; Shumway et al. 2021);
7. Whilst this paper has focused broadly on public and private land tenures generally, leasehold land in Australia raises particular complexities that is an important area for future research.

These challenges to coastal ecosystem restoration span those that may be implemented immediately at the project level (e.g. clarifying contractual arrangements), those that could navigated through minor policy adjustments (e.g. incentive schemes) and those that require major legislative reform (e.g. changing legal boundary rules). The rapid deployment of coastal ecosystem restoration would be aided by action on all recommendations irrespective of ease of implementation. Given the global impetus to restore nature, including coastal wetlands, this should be a priority area for governments.

Further, it should be noted that although this paper focuses on Australia, land tenure issues are not unique to the Australian context, and have hindered ecosystem restoration in other countries (Chazdon et al. 2017). Internationally, there is evidence of mangrove restoration projects which have been implemented low in the intertidal zone to avoid tenure disputes, which has limited the project’s success due to the inappropriate location (Lovelock and Brown 2019). Other studies have observed issues with tenure and communal ownership, access and management regimes (Asante et al. 2017; Friess et al. 2016). Thus this paper is intended to contribute to broader international conversations regarding land tenure, ownership and use and coastal wetland restoration.

## Supplementary information

Supplementary Material 1

Supplementary Material 2

## Footnotes

1 *Land Title Act 1994* (Qld) ss 97N-97U; *Conveyancing Act 1919* (NSW) Part 6, Division 4; *Climate Change Act 2010* (Vic) ss 3B, 26, 27-39; *Forestry Rights (Registration) Act 1990* (Tas); *Forest Property Act 2000* (SA); *Carbon Rights Act 2003* (WA).
