# Supplementary material for: Land Tenure, Ownership and Use as Barriers to Coastal Wetland Restoration Projects in Australia: Recommendations and Solutions
Source: Environ Manage. 2023 Apr 3;72(1):179–89. doi: 10.1007/s00267-023-01817-w (PMC10220139; doi:10.1007/s00267-023-01817-w)
Supplement: Supplementary file 1 — Supplementary Material 1 [file 267_2023_1817_MOESM1_ESM.docx]

Legislative review of law for restoration projects: (b) revegetation and (c) clearing vegetation for restoration.

**QUEENSLAND**

| **Activity Type** | **Legislation / Policy** | **Detail** |
| --- | --- | --- |
| Revegetation of marine plants | *Fisheries Act 1994* (Qld) | If revegetation disturbs existing marine plants (removal, destruction or damage to marine plants) a fisheries permit is required. |
|  | *Planning Act 2016* (Qld) (Schedule 7)  Accepted development requirements for operational work that is the removal, destruction or damage of marine plants | In some circumstances, notification to department required but no permit: captures certain rehabilitation works involving the use of mangrove seeds and propagules for mangrove rehabilitation within or immediately adjacent to the developer’s property (owned or leased) for both public and private purposes. Must be   - Within 100km of the rehabilitation site - By hand - Using unattached seeds/propagules   Under an endorsed project plan (with Fisheries Qld) or as part of an environmental offset. |
|  | *Planning Act 2016* (Qld) (Schedule 10)  State Code 11 | Permit required for restoration; activity must achieve performance criteria in State Code 11 to be granted a permit (including sourcing plants locally for revegetation). Restoration must not   - compromise condition of fish habitats or fisheries productivity; or - substitute a particular fish habitat for another type of habitat, for example, creation of mangrove communities from other tidal fish habitats; or - substitute a natural fish habitat for artificial fish habitat; or - deliver fish habitats that are likely to be regularly disturbed, such as through predictable sediment removal or maintenance dredging; or - deliver fish habitats that will predictably be at a high risk of contamination and/or further disturbance.   For revegetation, marine plants used must be sourced within 100km of site (PO31/AO31). |
| Clearing marine plants | *Fisheries Act 1994* (Qld) | Removal, destruction or damage to marine plants generally requires a fisheries permit. |
|  | *Planning Act 2016* Qld) (Schedule 7)  Accepted development requirements for operational work that is the removal, destruction or damage of marine plants | In some circumstances, notification to department required but no permit: captures certain rehabilitation works involving the removal of mangrove seeds and propagules for mangrove rehabilitation within or immediately adjacent to the developer’s property (owned or leased) for both public and private purposes. Must be   - Within 100km of the rehabilitation site - By hand - Using unattached seeds/propagules - Under an endorsed project plan (with Fisheries Qld) or as part of an environmental offset. |
|  | *Planning Act 2016* (Qld) (Schedule 10)  State Code 11 Removal, destruction or damage of marine plants | Otherwise, removal, destruction or damage requires a permit. SARA will assess whether the activity meets performance outcomes, which include that restoration does not -   - compromise condition of fish habitats or fisheries productivity; or - substitute a particular fish habitat for another type of habitat, for example, creation of mangrove communities from other tidal fish habitats; or - substitute a natural fish habitat for artificial fish habitat; or - deliver fish habitats that are likely to be regularly disturbed, such as through predictable sediment removal or maintenance dredging; or - deliver fish habitats that will predictably be at a high risk of contamination and/or further disturbance. |
| ***Potentially incidentally applicable legislation*** | | |
| *Nature Conservation Act 1992* (Qld) | | If activities result in “take” of a protected plant (which includes a range of interventions including removing or injuring plants) then a permit may be required under this Act. “Protected plants” do not include mangroves but may include other plants (non-marine) incidentally occurring at proposed restoration site. |
| *Coastal Protection and Management Act 1995* (Qld)  *Coastal Protection and Management Regulation 2017.* | | Act applies to works on coast (defined coastal management district, where specific coastal provisions apply under the planning regime).  Permit is required to damage or remove vegetation on State coastal land above the high-water mark (some exceptions relating to work falling under accepted development in planning framework). “Tidal works” is a broad term for activities on this land which require permission. Development may require assessment by the SARA.  Definition of state coastal land excludes freehold land / land contracted to be granted in fee simple by the State, and land subject to a lease or licence issued by the state (CMPA s17). |
| *Planning framework: SDAP State Code 9 – Great Barrier Reef wetland protection areas* | | Additional requirements may apply for activities in this district. |

**NEW SOUTH WALES**

| **Activity Type** | **Legislation / Policy** | **Detail** |
| --- | --- | --- |
| Revegetation of marine vegetation | *Fisheries Management Act 1994* (NSW)  *Environmental Planning and Assessment Act 1979* – integrated development | Harm to marine vegetation (even incidentally) will require a fisheries permit, including undertaking any prescribed activities.   - Harm: ‘to gather, cut, pull up, destroy, poison, dig up, remove, injure, prevent light from reaching or otherwise harm the marine vegetation’ - Prescribed activities include driving or operating a vehicle over marine vegetation. |
|  | *Coastal Management Act 2016* (NSW)  Coastal Management SEPP 2018 | Works in the coastal zone, specifically coastal wetlands and littoral rainforests area, require consent (also requires the submission of an environmental impact statement): SEPP cl 10(1). Harm to marine vegetation is explicitly identified as requiring consent in the SEPP cl 10(1)(b), as is environment protection works cl 10(1)(c)(iv).  Some exemptions for public authorities undertaking restoration works within coastal management programs: SEPP cl 10(2). |
|  | *Local Land Services Act 2013* (NSW)  Coastal Management SEPP 2018 | Note that any incidental clearing of “native vegetation” (which is *not* marine vegetation) in national park estate and other conservation areas (s60A(b)) and rural land (s60A(a)) will also require development consent (s60ZF), unless completed by a landholder under a land management (native vegetation) code (in certain areas) (s60S).  If undertaken in a coastal wetlands and littoral rainforests area, consent always required: cl 10(1)(a) Coastal Management SEPP. |
| Clearing marine vegetation | *Fisheries Management Act 1994* (NSW)  *Environmental Planning and Assessment Act 1979* (NSW) – integrated development | Clearing marine vegetation will meet the definition of “harm” to marine vegetation, requiring a fisheries permit, including if proponent undertakes any prescribed activities.   - Harm: ‘to gather, cut, pull up, destroy, poison, dig up, remove, injure, prevent light from reaching or otherwise harm the marine vegetation’ - Prescribed activities include driving or operating a vehicle over marine vegetation. |
|  | *Coastal Management Act 2016* (NSW)  Coastal Management SEPP 2018 | Works in the coastal zone, specifically coastal wetlands and littoral rainforests area, require consent (also requires the submission of an environmental impact statement): SEPP cl 10(1). Harm to marine vegetation is explicitly identified as requiring consent in the SEPP cl 10(1)(b), as is environment protection works cl 10(1)(c)(iv).  Some exemptions for public authorities undertaking restoration works within coastal management programs: SEPP cl 10(2). |
|  | *Local Land Services Act 2013* (NSW)  Coastal Management SEPP 2018 | Note that any incidental clearing of “native vegetation” (which is *not* marine vegetation) in national park estate and other conservation areas (s60A(b)) and rural land (s60A(a)) will also require development consent (s60ZF), unless completed by a landholder under a land management (native vegetation) code (in certain areas) (s60S).  If undertaken in a coastal wetlands and littoral rainforests area, consent always required: cl 10(1)(a) Coastal Management SEPP. |
| ***Potentially incidentally applicable legislation*** | | |
| *Crown Lands Management Act 2016* | | Applies to works on Crown land, and may require additional approvals or consultation. |

**VICTORIA**

| **Activity Type** | **Legislation / Policy** | **Detail** |
| --- | --- | --- |
| Revegetation of marine vegetation | *Planning and Environment Act 1987* (Vic) (s47)  Victoria Planning Provision | If revegetation incidentally removes, destroys or lops native vegetation, permit is required: 52.17-1 VPP. Exceptions include (52.17-7):   - For conservation work to the minimum extent necessary, with approval from the DELWP Secretary.   On Crown land (by or on behalf of several public authorities) in accordance with Procedure or with written permission. |
|  | *Marine and Coastal Act 2018* (Vic)  Marine and Coastal Policy 2020 | Works on marine and coastal Crown land require permission (and works include removing vegetation and topsoil). |
| Clearing marine vegetation | *Planning and Environment Act 1987* (Vic) (s47)  Victoria Planning Provision | A permit is required to remove, destroy or lop native vegetation, including dead native vegetation: 52.17-1 VPP. Exceptions include (52.17-7):   - For conservation work to the minimum extent necessary, with approval from the DELWP Secretary. - On Crown land (by or on behalf of several public authorities) in accordance with Procedure or with written permission. |
|  | *Marine and Coastal Act 2018* (Vic)  Marine and Coastal Policy 2020 | Works on marine and coastal Crown land require permission (and works include removing vegetation and topsoil). |
| ***Potentially incidentally applicable legislation*** | | |
| *Water Act 1989* (Vic) | | *Conditions may be placed on licences to take / use water from water sources in relation to ‘in-stream uses’ which include maintenance of aquatic, riparian, floodplain or wetland ecosystems.* |
| *Heritage Rivers Act 1992* (Vic) | | *If in an area declared as a “heritage river area” or “natural catchment area” then the ‘clearing of indigenous flora’ is prohibited.* |
| *National Parks Act 1975* (Vic) | | *If in a national park, additional requirements may apply.* |

**SOUTH AUSTRALIA**

| **SOUTH AUSTRALIA** | | |
| --- | --- | --- |
| **Activity Type** | **Legislation / Policy** | **Detail** |
| Clearing marine plants | *Native Vegetation Act 1991* (SA) | Clearing of native vegetation (which includes plants growing in or under waters of the sea) generally requires consent from the Native Vegetation Council.  For privately owned land, the landowner must make the application for consent. If the land is held from the Crown under a miscellaneous lease, the Minister for Environment and Conservation must make the application. |
|  | South Australia Planning and Design Code  *Planning, Development and Infrastructure (General) Regulations 2017* (SA) | Where a development involves the clearing of native vegetation on land within the Native Vegetation Overlay or State Significant Native Vegetation Overlay, depending on the category of clearance this may require referral to the Native Vegetation Council. |
|  | *Fisheries Management Act 2007* (SA) | A permit is required to remove or interfere with aquatic or benthic animals or plants (which includes mangroves) in, or disturb the bed of any, waters forming part of an aquatic reserve (s 77). |
| Revegetation of marine plants | *Native Vegetation Act 1991* (SA) | An owner of land can submit a proposal to the Native Vegetation Council regarding the revegetation of the land with plants of one or more species indigenous to the local area, that will be representative of a naturally occurring plant community (s 23F).  Depending on the tenure, the ‘owner’ may be the holder in fee simple, a lessee or the Minister for Environment and Conservation. |
|  | *Coast Protection Act 1972* (SA) | The Coast Protection Board is authorised to carry out any works necessary or expedient to restore any damage to the coast resulting from a storm or pollution, which may capture revegetation of marine plants (s 21).  The Board can, with the Minister’s approval, acquire any land constituting part of the coast (s 22). When seeking to enter private land, the Board must give the occupier or owner 7 days’ notice of intention to enter (s 23(2)). |
|  | Note that revegetation which incidentally involves interference or removal of existing vegetation will invoke the same legislative provisions outlined above concerning the clearing of vegetation.  In particular, revegetation that interferes with the bed of any waters in an aquatic reserve will require permission under the *Fisheries Management Act 2007* (SA) (see above). Similarly, revegetation or restoration involving filling of material (such as soil etc) within coastal land may trigger development controls under the *Planning, Development and Infrastructure Act 2016* (SA) (see below). | |
| ***Potentially incidentally applicable legislation*** | | |
| *Planning, Development and Infrastructure Act 2016* (SA) | | Development approval is required for works involving excavation and filling of a volume of material exceeding 9m^3^ within coastal land, or within 3 nautical miles seaward of the coast. |
| *Wilderness Protection Act 1992* (SA) | | Permission is required for a person to destroy or damage native plants in a wilderness protection area or zone, or on land acquired by the Minister for Environment and Water (s 27). |
| *Wilderness Protection Regulations 2021* (SA) | | The Director of National Parks and Wildlife’s permission is required for a person to:   - take an exotic plant that is growing in a wilderness protection area or wilderness protection zone (r 24(1)); - bring a plant into a wilderness protection area or wilderness protection zone (r 24(2)); and - remove, dig or otherwise intentionally disturb dead vegetation or soil in a wilderness protection area or wilderness protection zone (r 25). |

**WESTERN AUSTRALIA**

| **WESTERN AUSTRALIA** | | |
| --- | --- | --- |
| **Activity Type** | **Legislation / Policy** | **Detail** |
| Clearing marine plants | *Environmental Protection Act 1986* (WA) | A clearing permit is generally required for the clearing of native vegetation (indigenous aquatic or terrestrial vegetation), to be obtained by the owner of the land. Depending on the tenure, the relevant ‘owner’ of the land may be:   - the holder of freehold title; - the person entitled to the benefit of an agreement with the Crown to alienate the land; - the lessee; or - the public authority with care, control or management of the land (or the Crown if there is no such authority) (s 51A).   Clearing in contravention of a soil conservation notice or on land that is the subject of a conservation covenant or environmental protection covenant is prohibited, regardless of if a clearing permit is held (see s 51D(3)). |
|  | *Biodiversity Conservation Act 2016* (WA) | The taking of flora by a person on Crown land is prohibited, unless the person holds a clearing permit or other qualifies for an exemption under the *Environmental Protection Act 1986* (WA). However, for private land, the owner or occupier may take flora on that land (s 171(1)–(2)).  The taking of threatened flora on both Crown and private land requires authorisation by the Minister under s 40 (s 173). |
| Revegetation of marine plants | Revegetation which incidentally involves interference or removal of existing vegetation will likely invoke the same legislative provisions outlined above concerning the clearing of vegetation.    See particularly the *Waterways Conservation Act 1976* (WA) regarding disturbance of waterbeds, noted below in the ‘potentially incidentally application legislation’ section. | |
| ***Potentially incidentally applicable legislation*** | | |
| *Waterways Conservation Regulations 1981* (WA) | | For waters to which s 48 of the *Waterways Conservation Act 1976* (WA) applies, a person is prohibited (except on the Minister’s behalf) from disturbing the bed, banks or foreshore of any waters so as to endanger their stability or any vegetation present (r 8(1)(f)). |
| *Conservation and Land Management Act 1984* (WA) | | A person cannot take, destroy or otherwise interfere with any flora in a marine nature reserve, marine park or marine management area without:   - a licence granted under the *Conservation and Land Management Regulations 2002* (WA); - a licence granted under the *Biodiversity Conservation Regulations 2018* (WA); or - an authorisation given under section 40 of the *Biodiversity Conservation Act 2016* (WA). |

**NORTHERN TERRITORY**

| **NORTHERN TERRITORY** | | |
| --- | --- | --- |
| **Activity Type** | **Legislation / Policy** | **Detail** |
| Clearing marine plants | *Planning Act 1999* (NT)  NT Planning Scheme | A development permit is required for the clearing of native vegetation (terrestrial and inter-tidal flora indigenous to the Northern Territory, including mangroves) of more than one hectare in aggregate of land, where the land is within the *Clearing of Native Vegetation Overlay* or *Restricted Clearing of Native Vegetation Overlay.*  The *Clearing of Native Vegetation Overlay* applies to the following Zones:   - Rural Residential (RR); - Rural Living (RL); - Rural (R); - Horticulture (H); - Agriculture (A); - Community Purpose (CP); - Conservation (CN); - Restricted Development (RD); - Water Management (WM); and - Unzoned Land. |
|  | *Water Act 1992* (NT) | A permit is required to engage in conduct that interferes with a waterway (s 40). Such conduct includes causing a material change to the shape, flow volume or direction of a waterway, or altering the stability of the bend or banks of a waterway by removing vegetation. |
| Revegetation of marine plants | Note that revegetation which incidentally involves interference or removal of existing vegetation may invoke the same legislative provisions outlined above concerning the clearing of vegetation. See particularly the interfering with a waterway provisions in the *Water Act 1992* (NT). | |
| ***Potentially incidentally applicable legislation*** | | |
| *Territory Parks and Wildlife Conservation Act 1976* (NT) | | A permit is required to take or interfere with protected wildlife, meaning wildlife that is in a park, reserve, sanctuary, wilderness zone or area of essential habitat (s 55).  ‘Wildlife’ includes plants that are indigenous to the Australian coastal sea or the sea-bed and subsoil beneath that sea. |
| *Fisheries Act 1988* (NT) | | A licence must be obtained to take fish or aquatic life for the purpose of sale or aquaculture (s 10). ‘Aquatic life’ includes any species of plant (including seaweeds, sea-grasses and algae) that must inhabit water, but excludes aquatic life declared by the Minister in the Gazette. |

**TASMANIA**

| **TASMANIA** | | |
| --- | --- | --- |
| **Activity Type** | **Legislation / Policy** | **Detail** |
| Clearing marine plants | *Forest Practices Act 1985* (Tas) | A certified forest practices plan is required for the responsible person over land to clear and convert a threatened native vegetation community on that land (s 17(4)). ‘Clearance and conversion’ means the deliberate removal of all or most of the threatened native vegetation community on land, and then:   1. leaving the land in an unvegetated state; 2. replacing the threatened native vegetation with another community of native vegetation, non-native vegetation, agricultural works or residential, commercial or other non-agricultural development; or 3. doing a combination of things in (a) and (b).   Certification of forest practices plans is given by the Forest Practices Authority upon application. The responsible person will generally be the owner of the land. |
|  | *Crown Lands Act 1976* (Tas) | A person requires authorisation by the Minister for Parks to cut, remove, take or damage any trees or vegetation on Crown land or assigned land (s 46(1)(e)). |
|  | *Land Use Planning and Approvals Act 1993* (Tas)  Tasmania Planning Scheme – State Planning Provisions | Subject to the relevant planning scheme in place, a development permit may be required to clear vegetation. Relevantly, ‘works’ is defined to include any change to land topography, including the removal of vegetation or topsoil.  However, clearance and conversion of a threatened native vegetation community carried out under a certified forest practices plan will not require a separate development permit (State Planning Provisions, Table 4.4.1). |
| Revegetation of marine plants | *Forest Practices Act 1985* (Tas) | As noted, clearance and conversion of threatened native vegetation communities captures both removal and revegetation. A forest practices plan must be prepared by the responsible person of the land (generally the owner) and certified by the Forest Practices Authority. |
|  | *Land Use Planning and Approvals Act 1993* (Tas)  Tasmania Planning Scheme – State Planning Provisions | A development permit is not required for some vegetation rehabilitation works. This involves the planting, clearing or modification of vegetation for the removal or destruction of declared weeds or environmental weeds listed under a strategy or management plan approved by council (Table 4.4.3). |
| ***Potentially incidentally applicable legislation*** | | |
| *None to note.* | | *N/A* |
