# Supplementary material for: Land Tenure, Ownership and Use as Barriers to Coastal Wetland Restoration Projects in Australia: Recommendations and Solutions
Source: Environ Manage. 2023 Apr 3;72(1):179–89. doi: 10.1007/s00267-023-01817-w (PMC10220139; doi:10.1007/s00267-023-01817-w)
Supplement: Supplementary file 2 — Supplementary Material 2 [file 267_2023_1817_MOESM2_ESM.docx]

Legislative review of law for restoration projects: (a) removal of a waterway barrier, or restoring hydrology

| Legislation | | Administering body | Detail |
| --- | --- | --- | --- |
| QUEENSLAND | | | |
| *Coastal Protection and Management Act 1995* (Qld);  *Coastal Protection and Management Regulation 2017* (Qld);  *Planning Act 2016* (Qld);  *Planning Regulation 2017* (Qld) | Department of State Development, Infrastructure, Local Government and Planning (PA and PR); Department of Environment and Science (CPMA and Reg)  [Local Councils] | | **Consent requirements:** in Queensland, operational works that are tidal works (which includes the construction and demolition of seawalls, breakwaters, groynes and embankments, works in tidal water associated with such construction or demolition, and reclamation of land under tidal water) are classified as code assessable development. For ‘prescribed tidal works’ (which is defined in the *Coastal Protection and Management Regulation 2017* to mean all tidal works, but excluding those carried out in State managed boat harbours or involving construction of new port authority structures or alterations to navigational channels), the local government is the assessment manager.  In most circumstances, development of this type (operational works that is tidal works) will also require referral. The following must be referred to the State Assessment and Referral Agency (**SARA**) to be assessed against the State Development Assessment Provisions (**SDAP**) (or the Gold Coast Waterways Authority against the *Gold Coast Waterways Authority Act 2012*, if it is carried out in Gold Coast waters):   - tidal works; - disposing of dredge soil or other solid waste material in tidal water; - reclaiming land under tidal water; or - constructing a canal, if the canal relates to reconfiguring a lot.   **Notice requirements:** written notice to neighbouring landholders is not required. In addition, public notification is not required by the applicant in respect of such tidal works (as they are code assessable development).  **Decision-making requirements:** development approval is discretionary.  **Consideration of adverse impacts:** the local government determining an application involving prescribed tidal works must do so against the *Code for assessable development that is prescribed tidal works*, which includes performance criteria such as:   - excavation and filling is carried out only to the extent necessary, and does not have a significant impact on natural features etc (PO7.1); - adverse impacts to vegetation are limited; (PO7.2); - revetments and seawalls are designed to support their intended loads, having regard to their relevant loading matters and intended design life (PO19.1).   When assessing a referred application, SARA must consider various performance criteria which includes (per the SDAP, State code 8: Coastal development and tidal works)):   - erosion control structures are only constructed where there is an imminent threat to buildings or infrastructure of value, and there is no feasible option for either beach nourishment or relocation/abandonment of structures (PO9); - erosion control structures minimise interference with coastal processes (PO10); - all dredging is safe and is supported by a monitoring and management plan that protects the marine environment (PO21); - development does not involve reclamation of land below tidal water, other than for coastal-dependent development, public marine development  or coastal protection work (PO22). |
| *Water Act 2000* (Qld); *Planning Regulation 2017* (Qld) | Department of Environment and Science | | A licence is required to take or interfere with water. ‘Interference’ with the flow of water in a watercourse includes diverting the course or water in a watercourse outside of its bed and banks.  Projects involving the construction or modification of levees may also, depending on the category of levee, require development approval. Construction of a new category 1 levee (or modification of an existing levee, where the resulting levee falls under category 1) is accepted development not requiring approval. However, construction of new category 2 levees (or modification, where the resulting levee falls under category 2) requires code assessment by the local government, and construction of new category 3 levees (or modification where the resulting levee falls under category 3) necessitates impact assessment, which will require the preparation of an EIS (see *Water Regulation 2016* (Qld)).  ‘Modification’ of an existing levee means to raise or lower the levee’s height, extend or reduce its length, or to make another change to the levee that affects the flow of water. |
| *Fisheries Act 1994* (Qld) | Department of Agriculture and Fisheries | | A fisheries permit is needed to remove, destroy or damage a marine plant. |
| *Nature Conservation Act 1992* (Qld); *Nature Conservation (Plants) Regulation 2020* (Qld) | Department of Environment and Science | | A clearing permit is required to clear protected native plants. |
| **NEW SOUTH WALES** | | | |
| *Coastal Management Act 2016* (NSW); State Environmental Planning Policy (Coastal Management) 2018; *Environmental Planning and Assessment Act 1979* (NSW); Local Environment Plans | | Department of Planning, Industry and Environment  [Local Councils & Regional Planning Panels] | **Consent requirements:** consent must be obtained to undertake “coastal protection works” (defined to include seawalls). All development by individuals in coastal wetlands and littoral rainforests planning zone requires consent (unless done on behalf of a public authority) (includes earthworks [depositing material on land] and constructing a levee). Local Environment Plans also prescribe additional development controls, specific to each council area.  Applications for work in the coastal wetlands and littoral rainforests zone must be accompanied by an EIS.  **Notice requirements:** written notice not required to neighbouring landholders. Public notification is required for works in this zone (on consent authority’s website and on land the subject of the proposal). A person may make a submission by way of objection and if dissatisfied with determination to grant consent, may appeal to NSWLEC.  **Decision-making requirements:** development consent is discretionary.  **Consideration of adverse impacts:** in determining applications the consent authority must take into account:   - Likely environmental impacts on both natural and built environment, and social and economic impacts in the locality; - Any submissions made.   In the coastal wetlands and littoral rainforests area, the consent authority must not grant consent unless satisfied that sufficient measures have been, or will be, taken to protect, and where possible enhance, biophysical, hydrological and ecological integrity.  Consent must be refused only where it would contravene the Acts, or environmental planning instruments, and other limited circumstances, where works: will or will be likely to unreasonably limit public access; or pose a threat to public safety. |
| *Fisheries Management Act 1994* (NSW); *Environmental Planning and Assessment Act 1979* (NSW); Policy and Guidelines for Fish Habitat Conservation and Management (Update 2013) | | Department of Planning, Industry and Environment (integrated development applications); Department of Primary Industries | Consent is required for “dredging” activities (which means excavating water land or moving material on or from water land). Permit applications require inclusion of an aquatic habitat assessment and aquatic fauna assessment, as well as potentially a detailed aquatic survey depending on works’ impact (which may involve hydrological modelling).  Harm to marine vegetation (in the course of works) requires a fisheries permit. |
| *Marine Estate Management Act 2014; Marine Estate Management (Management Rules) Regulation 1999;* Aquatic Reserve Notification 2015 | | Department of Primary Industries | Consent is required to undertake work within a marine park, or aquatic reserve. |
| **VICTORIA** | | | |
| *Marine and Coastal Act 2018* (Vic); *Marine and Coastal Policy 2020; Planning and Environment Act 1987* (Vic); Victoria Planning Provisions (VPPs); local planning schemes | | Department of Environment, Land, Water and Planning;* local councils [specific requirements are localised in VIC]  *Consent from the Minister for Energy, Environment and Climate Change required prior to the planning body providing consent for works in this zone. | **Consent requirements:** Any use, development or undertaking of works on marine and coastal Crown land requires consent. Permit requirements on other public or private land depend on the individual zone of the land, though the Marine & Coastal Policy applies to the entire marine and coastal environment to guide policy decisions.  **Notice requirements:** [where planning scheme requires consent] notice must be given to the owners and occupiers of allotments or lots adjoining the land to which the application applies (unless the responsible authority is satisfied that the granting of a permit would not cause material detriment to any person). Any person affected by the grant of a permit may object to the development.  **Decision-making requirements:** consent is discretionary.  **Consideration of adverse impacts:** in making a decision, the responsible authority must consider:   - any significant effects the use or development may have on the environment; and - any significant social and economic effects. - If there are objections, the responsible authority must consider them, though they are not required to reject the application on that basis.   Only development that will provide significant net community benefit should be approved in marine and coastal Crown land (structures with no public use benefit are prohibited). |
| *Planning and Environment Act 1987* (Vic); Victoria Planning Provisions (VPPs); local planning schemes | | Department of Environment, Land, Water and Planning; local councils | In many zones, if the works have the effect of clearing native vegetation, a permit to do so is required. |
| *Flora and Fauna Guarantee Act 1988* (Vic) | | Department of Environment, Land, Water and Planning | A permit is needed if the works have the impact of “taking” (defined as killing, injuring, disturbing, or collecting), keeping, moving or processing protected flora. |
| **WESTERN AUSTRALIA** | | | |
| *Planning and Development Act 2005* (WA); State Planning Policy 2.6; *Planning and Development (Local Planning Schemes) Regulations 2015* (WA); local planning schemes | | Department of Planning, Lands and Heritage  Local Councils | **Consent requirements:** As with Victoria, land use planning and development controls in WA are created at the local government level. This means that whether development (which includes the demolition and construction of structures on land, and the carrying out of excavation works) is permitted will depend on the local planning scheme. Where the local planning scheme provides that development must not be commenced without consent, approval must be obtained.  Regarding such schemes, the State Planning Provisions (which must be considered by local governments when preparing or amending a local planning scheme) strongly warrant against the approval of new coastal protection works.  In addition, the *Planning and Development (Local Planning Schemes) Regulations 2015* prescribe several ‘deemed provisions’ which may be enforced as part of every local planning scheme. These include notice requirements and assessment considerations (see below).  **Notice requirements:** while notice obligations will depend on each local planning scheme, the deemed provisions provide that for development applications that are not ‘complex applications’ nor identified in the relevant scheme as a type of application requiring advertisement, the local government may choose to advertise the application by:   - publishing the application documents and accompanying material; and - giving notice of the proposed development to owners and occupiers in the vicinity who, in the local government’s opinion, are likely to be affected by the granting of development approval.   **Decision-making requirements:** development approval is discretionary.  **Consideration of adverse impacts:** the deemed provisions state that the local government assessing the application must have due regard to different matters, including:   - the amenity of the locality, including environmental impacts of the development; - the likely effect of the development on the natural environment or water resources and any means that are proposed to protect or to mitigate impacts on the natural environment or the water resource; - the suitability of the land for the development taking into account the possible risk of flooding, tidal inundation, subsidence, landslip, bush fire, soil erosion, land degradation or any other risk; - any submissions received on the application. |
| *Environmental Protection Act 1986* (WA) | | Department of Water and Environmental Regulation | A person must obtain a clearing permit issued by the Department of Water and Environmental Regulation in order to cause or allow the clearing of native vegetation. |
| *Waterways Conservation Act 1976* (WA)*; Waterways Conservation Regulations 1981* (WA) | | Department of Water and Environmental Regulation | A person must apply to the Minister for Water for a licence to construct a retaining wall at the bank of any waters and to dredge in or reclaim waters. |
| **SOUTH AUSTRALIA** | | | |
| *Planning, Development and Infrastructure Act 2016* (SA); *Planning, Development and Infrastructure (General) Regulations 2017* (SA); SA Planning and Design Code | | Department of Planning, Transport and Infrastructure; Coast Protection Board  [Local Councils] | **Consent requirements:** local government approval must be obtained to carry out ‘development’, which includes the construction of any coastal protection structure (anything designed to control coastal erosion), any excavation or filling on coastal land of more than 9m**^3^** of material, and the forming of a levee or mound greater than 3m above the ground. More generally, ‘development’ also includes the demolition and removal of structures.  The level of development assessment required for such works depends on the Zones (and Subzones, where applicable) in which the proposal is situated. These are defined in the new SA Planning and Design Code (**the Code**), which (following its completed rollout in early 2021) will replace all council development plans to be the single source of planning policy for assessing development applications. Under the Code, development may be categorised into one of the following categories, each with varying procedural implications:   1. Accepted (no planning consent required); 2. Deemed-to-satisfy (planning consent must be granted if it meets all deemed-to-satisfy criteria; no public notification required); 3. Restricted or impact assessed (assessed by SA Planning Commission; requires an EIS); 4. Performance assessed (development that is not classified as any of the other three types; will be assessed on its merits against the Code).   In addition, applications for specific types of development on land in the Coastal Areas Overlay under the Code (which covers the whole South Australian coastline, per the SA Property and Planning Atlas <https://train.sappa.plan.sa.gov.au/>) must be referred to the Coast Protection Board. The types of development requiring referral include (note the overlap with the definition of ‘development’ above):   - Excavation and/or filling where the total volume of material excavated and/or filled exceeds 9m3; - off-shore structures; - coast protection works; and - infrastructure within 100m landward of the mean high water mark.   After considering the application, the Board can direct the assessment authority (the local government) to refuse the application or, if it decides to approve, impose conditions. The purpose of this referral process is to provide expert advice and direction to the assessment authority on:   - the risk to development from current and future coastal hazards (including sea-level rise, coastal flooding, erosion, dune drift and acid sulfate soils) - coast protection works; - potential impacts from development on public access and the coastal environment.   **Notice requirements:** notice requirements will also vary depending on the category of development. While there are no notice obligations for applications for accepted and deemed-to-satisfy development, proponents of performance assessed and restricted/impact assessed development are required to give notice to:   - each owner and occupier of each piece of adjacent land; and - members of the public (by placing a notice on the relevant land).   It is most likely that the development considered here will be performance assessed development (or, in some circumstances, impact assessable). Therefore, these notice requirements will apply.  **Decision-making requirements:** development approval is discretionary.  **Consideration of adverse impacts:** the local government responsible for assessing the application must do so against Zone-specific performance criteria in the SA Planning and Design Code. All development considered here is likely to be in the Coastal Areas Overlay, which warrants assessment against various performance outcomes that include:   - effects on the marine and onshore coastal environment by pollution, erosion, damage or depletion (PO 4.1); - whether the development allows for ecological and natural landform adjustment to changing climatic conditions and sea levels by allowing landward migration of dunes, coastal wetlands, mangrove and samphire areas (PO 4.3).   Where a development application is referred to the Coast Protection Board, the Board will determine the application with reference to the *Policy on coast protection and new coastal development (1991)*. This states that the Board should not approve coastal protection works if they will lead to erosion of neighbouring land, loss of beach amenity, or other adverse environmental effects. |
| *Native Vegetation Act 1991* (SA) | | Native Vegetation Council | A consent must be obtained from the Native Vegetation Council to authorise clearance (killing or destruction, removal, and any other substantial damage, including the draining or flooding of land) of native vegetation. |
| *Environment Protection Act 1993* (SA) | | Environment Protection Authority | To undertake a ‘prescribed activity of environmental significance’, a person must obtain a licence from the Environment Protection Authority. Dredging is a prescribed activity of environmental significance. |
| *Fisheries Management Act 2007* (SA) | | Department of Primary Industries and Regions | A person requires a permit in order to engage in operations involving or resulting in the disturbance of the bed of any waters forming part of an aquatic reserve, or removal of or interference with aquatic or benthic animals or plants (which includes mangroves) of any waters that are part of an aquatic reserve.  An ‘aquatic reserve’ is any land and/or waters declared to constitute an aquatic reserve by the Governor. |
| **TASMANIA**  **Note: Tasmania is undergoing major planning reform to introduce the Tasmania Planning Scheme, which is scheduled to be fully implemented by end 2021 and introduces statewide Planning Provisions, implemented locally through Local Provisions Schedules.* In Burnie council area alone, the new reforms are in effect. All other council areas operate with interim planning schemes. This summary addresses the current provisions applying to most of the State. | | | |
| *Land Use Planning and Approvals Act 1993* (Tas); *Environmental Management and Pollution Control Act 1994* (Tas);  Local planning schemes (mostly interim) | | Department of Primary Industries, Parks, Water and Environment; Tasmanian Planning Commission; Local Councils | **Consent requirements:** Similarly to Victoria and Western Australia, land use planning is localised, and determined according to the zone in which works are proposed to be undertaken. Unless specifically declared exempt from permit requirements or explicitly identified as not needing a permit, any use or development requires a permit. There are limited exemptions (particularly in the coastal zone).  **Notice requirements:** for use or development where planning authority has discretion to refuse or permit, the planning authority must give notice to the owners and occupiers of all properties adjoining the land that is the subject of the application.  **Decision-making requirements:** consent is discretionary.  **Consideration of adverse impacts:** In making a decision, the Minister must have regard to the performance criteria in local planning schedules (currently interim); these generally relate to environmental (and other) impacts on the area.  If the activity will cause any environmental harm (defined as ‘any adverse effect on the environment, of whatever degree or duration), the *Environmental Management and Pollution Control Act 1994* will apply to require an environmental impact assessment (the extent of which is dependent on the extent of the harm). |
| *Nature Conservation Act 2002; Threatened Species Protection Act 1995; Threatened Species Protection Regulations 2016; National Parks and Reserves Management Regulations 2019* | | Department of Primary Industries, Parks, Water and Environment (Conservation Assessments, Natural and Cultural Heritage Division) | If the works make an impact that amounts to “taking” (which includes destroy or damage a plant, fruit, seed or other plant parts, or to capture, destroy or damage fauna or products of wildlife including nests and dens) threatened flora, fauna and products of wildlife, a permit may be required. |
| *Crown Lands Act 1979* | | Department of Primary Industries, Parks, Water and Environment; Tasmania Parks and Wildlife Service | Work undertaken on Crown land requires additional licences, including to take marine plants. |
| **NORTHERN TERRITORY** | | | |
| *Planning Act 1999* (NT) | | Department of Infrastructure, Planning and Logistics; Northern Territory Planning Commission | **Consent requirements:** consent is required for “excavation and fill” works (the removal or importation of material to, from or within a site that will change the ground level of the land).  **Notice requirements:** written notice to neighbouring landholders (land adjoining the land to which the development application relates) is required and they may give written submissions.  **Decision-making requirements:** consent is discretionary.  **Consideration of adverse impacts:** Applications require information concerning:   - suitability of the site for future use; - a hydrological assessment of upstream and downstream impacts; - a management plan to control erosion and sedimentation; and - measures to prevent the creation of mosquito breeding areas.   Even if the works do not meet the development requirements, a consent authority may still approve if consistent with the zone purpose and outcomes, and appropriate to the site. |
| *Planning Act 1999* (NT); Land Clearing Guidelines 2020 (NT) | | Department of Infrastructure, Planning and Logistics; Northern Territory Planning Commission | If works have the effect of clearing (removing, destroying, by any means) native vegetation, a development application may be required. |
| *Aboriginal Land Rights (Northern Territory) Act 1978* (Cth) | | Commonwealth Dept Attorney-General; Dept Prime Minister and Cabinet | Permits are necessary to enter (including to pass through) Aboriginal land. |
| *Territory Parks and Wildlife Act 1976* | | Department of Environment, Parks and Water Security | If there is the presence of threatened and/or significant species, an application may be required. This requires a biodiversity assessment. |
| *Soil Conservation and Land Utilisation Act 1969* | | Department of Environment, Parks and Water Security | If activity falls within a restricted area as declared under this Act, removal of any sand, gravel rock, clay or earth is prohibited, as is causing water to be drained over the area (e.g. Cox Peninsula Restricted Use Area). Sea wall construction in this area would be prohibited. |
